# Prevalence, incidence and residual risk of transfusion transmitted viruses (HBV, HCV and HIV infections) in Lithuanian blood donors from 2004 to 2018: The incidence/window-period model study

**DOI:** 10.1371/journal.pone.0246704

**Published:** 2021-02-19

**Authors:** Samanta Grubyte, Jurgita Urboniene, Laura Nedzinskiene, Auguste Jelinskaite, Kestutis Zagminas, Arvydas Ambrozaitis, Ligita Jancoriene

**Affiliations:** 1 Department of Public Health, Institute of Health Sciences, Faculty of Medicine, Vilnius University, Vilnius, Lithuania; 2 Clinic of Infectious Diseases and Dermatovenerology, Institute of Clinical Medicine, Faculty of Medicine, Vilnius University, Vilnius, Lithuania; 3 Center of Infectious Diseases, Vilnius University Hospital Santaros Klinikos, Vilnius, Lithuania; 4 Department of Anatomy, Histology and Anthropology, Institute of Biomedical Sciences, Faculty of Medicine, Vilnius University, Vilnius, Lithuania; Centre de Recherche en Cancerologie de Lyon, FRANCE

## Abstract

**Introduction:**

Estimation of hepatitis B virus (HBV), hepatitis C virus (HCV) and human immunodeficiency virus (HIV) transfusion risk in blood donors is essential for monitoring the safety of the blood supply and the impact of new screening tests. Due to improvements in donor selection and continuing progress in screening assays, residual risk of virus transmission has significantly decreased over the past years. It is not practical and sometimes even not possible to measure residual risk in blood donors directly and mathematical models are used. The aim of this study was to calculate the prevalence, incidence rates of HBV, HCV and HIV infections and analyse evolution of their transmission residual risk from 2004 to 2018 at the National Blood Center of Lithuania.

**Materials and methods:**

Data from the archives of the National Blood Center of Lithuania from 2004 to 2018 was retrospectively analysed. The residual risk was calculated for each virus and year by applying the incidence/window-period model suggested by World Health Organization. For the analysis of the residual risk yearly trends a linear regression was used.

**Results:**

A total of 754,755 blood donors and 1,245,568 donations were included in the analysis and represented a 2.06 donations per donor over 15 years. Average residual risk for HBV, HCV and HIV respectively was 570.04, 807.14 and 35.72 per 1,00,000 donations. During the study period, there was statistically significant downward trend in the residual risk for every analysed virus.

**Discussion:**

Residual risk of virus transmission has been steadily decreasing over past 15 years in Lithuanian donors, but the current risk remains quite high. It is difficult to establish how much the risk is affected by statistical assumptions or virus prevalence in general population. However, results of this study indicate the need of the population screening program of transfusion transmitted viruses.

## Introduction

Blood and blood components are donated, prepared and used by the healthcare services worldwide; therefore, blood product safety remains an important concern of blood collection centres. Blood safety issues are mostly caused by transfusion transmitted viruses (TTVs)–human immunodeficiency viruses Type I/ Type II (HIV), hepatitis B virus (HBV) and hepatitis C virus (HCV) [1–3]. The AIDS epidemic was certainly the greatest threat to the blood supply in the 20^th^ century and had a great impact on how donated blood is screened today [2]. Blood safety is closely related to prevalence of HBV and HCV infections in general population. As per results of chronic hepatitis C infection study, blood transfusion was one of the biggest risk factors of HCV infection before the introduction of innovative screening strategies [3]. According to the recent data from the European Centre for Disease Prevention and Control (ECDC), HBV, HCV and HIV are mostly spread during sexual intercourse or injecting drug use. However, one of the most-commonly reported routes for viral hepatitis transmission still is hospitals or other healthcare facilities [4–6]. Data of a review conducted in 2017 showed that the prevalence of HBsAg and anti-HCV among first-time blood donors ranged accordingly from 0.00 to 3.22% and from 0.00 to 2.17 in different European countries. It is worth to highlight, that the highest prevalence of these viruses among all the investigated countries was detected in Bulgaria, Romania, Greece, Latvia and Lithuania [7].

The keys of the blood safety strategies are: obtaining entire blood supplies through voluntary, non-remunerated and regular blood donors from low risk populations, introducing effective blood donor selection measures, screening all donated blood for the markers of TTVs with highly sensitive, specific assays [8–11]. The strict regulations on blood safety and advances in the laboratory testing techniques have significantly reduced risk of TTVs transmission through donations, but infectious diseases continue to pose a risk [4–6, 7, 12, 13]. At present, the transmission of TTVs in the blood donor population mostly occurs because donors are in the diagnostic window period; also, in rare cases, because of assay failures (when the viruses are undetected by the particular assay used) and when an inadequate quality management system is applied [12, 14–16]. The World Health Organization (WHO) defines the diagnostic window period (DWP) as the “time interval from the infection to the time point when a blood specimen from that infected person first yields a positive result in a diagnostic or screening assay for the agent”. The length of the DWP depends on the replication kinetics of the virus during the different stages of infection, the screening assay category, its sensitivity for the specific TTV. Importantly, the DWP consists of two phases: the eclipse period during which the virus has already infected the donor but is not yet detected in the blood, even by highly-sensitive laboratory tests (such as NAT); and the ramp-up phase during which the viral concentration increases exponentially in the blood until a peak in the virus number is reached (viraemic phase). The viraemic phase of the DWP (vDWP), when the virus is already present in the blood but is still not detected by screening assays, is important for ensuring blood safety. The probability of accepting a TTV infected donation from an asymptomatic donor and of missing this infection with the screening assay is described as a residual risk [17].

The most accurate data on the residual risk is obtained by screening and tracking all recipients of blood or its components, but this is not a practical solution as large sample sizes and long-term follow-ups are required. Also, the introduction of strict selection of donors and effective screening tests have significantly reduced residual risk of TTVs that it is almost impossible for prospective studies of transfusion recipients to give accurate estimates [12, 18, 19]. However, by knowing the factors affecting the TTVs transmission risk, mathematical models to calculate the residual risk are developed. In the case of our research, when only limited incidence data on the TTVs and no accurate data about the TTVs risk in first-time donors are available, the incidence /window-period model suggested by the WHO is the most suitable [17].

Although, residual risk of TTV transfusion was steadily decreasing in the past years, it still occurs and should be constantly monitored as part of the prevention strategies. By applying incidence/window-period model, the present analysis was carried out with the aims: 1. To calculate the prevalence and incidence rates of HBV, HCV and HIV infections in blood donors; 2. To describe the residual risk of transfusion transmitted HBV, HCV and HIV; 3. To compare changes of HBV, HCV and HIV infections residual risk over time in the blood donor population.

It is important to highlight that the data regarding the prevalence and incidence of HBV, HCV and HIV infections in Lithuania is scarce. To the authors’ knowledge, no research about the residual risk of TTVs transmission in Lithuanian donors have been previously published.

## Materials and methods

Data from the annual statistical reporting forms of the National Blood Center of Lithuania from 2004 to 2018 was retrospectively analysed and the prevalence, incidence rates of HBV, HCV, HIV infections, as well as the trends of their transmission residual risk were described. As per local legislations, this survey is not the subject of bioethical regulation, because generalized and anonymized data (as opposed to personal health records) has been used. The Lithuanian Bioethics Committee reviewed the methodology of this survey and deemed the investigation an evaluation of service, not requiring review by an ethics committee.

Blood or its products are collected and served to the Lithuania’s hospitals by the National Blood Center of Lithuania and two hospital-based centres. The National Blood Center of Lithuania covers approximately 67.5% of all donations collected in Lithuania [20]. As per local regulations, blood or its components can be donated by all healthy citizens aged from 18 to 65 years in Lithuania. Selection of blood donors consists of two steps: 1. Self-written questionnaire before donation and evaluation of the health status of a potential blood donor. It is assumed that donors understand the questions asked and that their answers are truthful. Donor questionnaire covers information about the current general health of the donor, as well as certain behaviours associated with TTVs; 2. Post-donation testing of blood or its components.

All the donations from donors who pass first step are then mandatorily tested with serological tests for surface antigen of HBV (HbsAg), antibodies of HCV (anti-HCV), HIV-I/HIV-II antibodies, HIV-I antigens (ag/anti-HIV) at the National Blood Center of Lithuania. Since 2005 nucleic acid testing (NAT)–HBV DNA, HCV RNA and HIV RNA—is performed for all the donations that test negative for HBV, HCV and HIV with serological tests. Donations made by first-time donors undergo NAT testing for every individual donation (ID-NAT), while donations made by repeat donors are tested in mini-pools of 6 donations (MP6-NAT). Donation samples that were part of reactive mini-pool are later tested individually. Only results of the NAT testing since 2012 are included in this study due to the specifics of the official statistical forms. If the serological or NAT tests are initially positive, confirmatory tests are performed for those donations.

The following serological tests were used at the National Blood Center of Lithuania during the study period: serological HbsAg, anti-HCV and anti-HIV immunoassays were performed on the Abbott AxSym (Abbott, Wiesbaden, Germany) for period from 2005 to 2008. Starting from 2008, HbsAg, anti-HCV and HIV Ag/Ab Combo were performed on the Abbott Architect system. NAT testing was performed using Procleix Ultrio reagents on the Procleix Tigris (Grifols, S.A., Spain) system from 2005 to 2009 and—Procleix Ultrio Plus reagents since 2010.

In the year 2003 the right to give non-remunerated donations of blood or its components was approved in Lithuania and number of non-remunerated donations has reached 100% in the National Blood Center of Lithuania in 2018.

### Definitions

In this study, first-time donors were those who had donated blood or its components for the first-time, and whose blood/plasma were tested for HBV, HCV and HIV without evidence of any prior donation or testing. Repeat blood donors were those who had donated blood or its components at least one time previously (not necessarily in the same year) and whose blood/plasma were tested for HBV, HCV and HIV. For the calculation of the residual risk, part of the repeat donors, those who had made HBV, HCV and HIV positive donation after having passed it in their previous donation, were defined as seroconvertive donors. Donations were considered positive if they were tested positive for HBV, HCV or HIV with serological or NAT tests and remained positive after confirmatory test. Particular donors were defined as first-time, repeat and seroconvertive using statistical forms provided by the National Blood Center of Lithuania.

The vDWP (the viremic phase of DWP) was defined, as the time period between the appearance of a viral particle in 20 ml of plasma and the point of the first detectability of the viral marker by the screening assay, as suggested in the WHO guidelines [17]. If certain assumptions are made, the length of the vDWP can be described for the different assay categories and viruses. Based on the strategies performed for TTVs screening at the National Blood Center of Lithuania, the duration of the shortest vDWP suggested by the WHO guidelines was chosen to calculate the residual risk (Table 1) [17].

**Table 1:** Length of vDWP for different TTV screening assays used at the National Blood Center of Lithuania.

TTV	Years	Screening assay used in Lithuania	Length of vDWP^1^ based on WHO guidelines (days)
HCV	2004–2018	Antibody EIA/CLIA	60
	2012–2018^2^	ID-NAT^4^	3
HIV	2004–2007	Antibody EIA/CLIA	21
	2008–2018^3^	Combo EIA/CLIA	16
	2012–2018^2^	ID-NAT^4^	4
HBV	2004–2018	Antigen EIA/CLIA	42
	2012–2018^2^	ID-NAT^4^	17

Length of vDWP for the assay categories used for the HBV, HCV and HIV screening at the National Blood Center of Lithuania during different study periods, based on recommendations of World Health Organisation *‘Guidelines on estimation of residual risk of HIV*, *HBV and HCV infections via cellular blood components and plasma’* [17].

CLIA—chemiluminescence immunoassay.

EIA- enzyme immunoassay.

MP- 6 –nucleic acid amplification technique in mini-pool of 6 donations.

vDWP—viraemic phase of diagnostic window period.

^1^The vDWP was described as the presence of one virus particle in 20 ml of plasma for packed red blood cells [17].

^2^ The number of seroconvertive donors was calculated as a sum of donors who made serologically or NAT positive donation for the period from 2012 to 2018.

^3^Since 2008 HIV antibody testing (used for period from 2005–2007) was started to combine with HIV-I Ag test for HIV testing.

^4^ The vDWP for the MP6-NAT is not available in the recommendations of WHO, but it is considered that the smaller mini-pool size, the shorter the vDWP is. NAT testing of repeat donors at the National Blood Center of Lithuania is composed of testing in mini-pools of six donations and individual testing. Bearing in mind complexity of NAT testing, we have used the shortest ID-NAT vDWP suggested by WHO for the particular virus during period from 2012 to 2018 [17].

### Statistical analysis

The prevalence of HBV, HCV and HIV was calculated by dividing the number of positive for the particular virus donations (serologically and NAT) in a calendar year by the total number of donations in that year and was expressed per 100,000 donations. Prevalence was calculated for 2-year intervals for period from 2004 to 2017.

The incidence of HBV, HCV and HIV was calculated by dividing the number of seroconvertive for the particular virus donors in a calendar year by the total number of repeat donors in that year and was expressed per 100,000 person years.

The HBV, HCV and HIV residual risk for seroconvertive donors was calculated multiplying the incidence by the length of the vDWP selected with respect to the assay used (as represented in Table 1) and was expressed as a fraction of year.

RR=Incidence×vDWP

In the case of HBV, an adjustment factor that takes into account the cases that are undetected due to the transient nature of the antigenaemia and the viremia of HBV was applied. In order to adjust for this issue, we calculated the probability (P) of missing a detectable HBV infection in repeat donors by the respective screening assays, taking into consideration the following assumptions:

- 5% of HBV infected people become chronic carriers and express HBsAg and HBV-DNA permanently; thus, once the window period has passed these infected donors are detected by serology and by NAT.
- 25% of HBV infected people present a primary antibody response without expressing HBsAg and this is detected by a serological assay. 70% present a transient antigenaemia; thus, their probability of being detected by a serological assay is estimated by the HBsAg detection period divided by the inter-donation interval (IDI).
- 95% of HBV infected people present a transient viremia; thus, their probability of being detected by NAT is estimated by the HBV-DNA detection period divided by the IDI [17].

The length of the IDI is determined by the donation frequency of repeat donors, and it was calculated by dividing the observation period of one calendar year (365 days) by the average number of donations per repeat donor.

$$\text{IDI}\;=\;\frac{365\;\text{days}}{\text{average}\;\text{number}\;\text{of}\;\text{donations}\;\text{per}\;\text{repeat}\;\text{donor}}$$

For the different diagnostic assays, a mean detection period for the HBV marker is defined. We used the detection period of 60 days for the HBsAg assay and 90 days for HBV-DNA NAT, as suggested by the WHO [17].

$$\text{P}\;(\text{HBsAg})\;=\;70\%\text{x}\frac{\text{HBsAg}\;\text{detection}\;\text{period}}{\text{IDI}}+5\%$$

$$\text{P}(\text{NAT})\;=\;95\%\text{x}\frac{\text{HBV}-\text{DNA}\;\text{detection}\;\text{period}}{\text{IDI}}+5\%$$

Finally, the HBV incidence adjustment factor was calculated by dividing 100% from the probability of missing detectable HBV with a respective diagnostic assay (100%/P).

It is not possible to calculate the incidence rate among first-time donors directly, because it is unknown if a positive result corresponded to a new case or to a prevalent chronic case. Based on the WHO recommendations, an adjustment factor of 3 was used to calculate the incidence rate among first-time donors.

The overall prevalence and residual risk for each TTV and year was calculated as a weighted mean of the prevalence/residual risk of the repeat donors’ and first-time donors’ donations.

For the analysis of the yearly trends in the TTVs, a linear regression was used

The statistical calculations were performed using Microsoft Office Excel, WinPepiVersion11.65 and IBM SPSS Statistics Version 20.0. A p value < 0.05 has been considered to be statistically significant.

## Results

From January 2004 to December 2018, 754,755 blood donors in total made 1,245,568 donations in the National Blood Center of Lithuania. On average, 83,038 donations were made each year. 23.51% (292,827) of the donations were made by first-time blood donors and 76.49% (952,741) were made by repeat donors. Repeat donors composed 61.20% (from 47.38 to 71.17% per year) of the total donors (Table 2).

**Table 2:** Number of blood donors and donations in the Lithuanian National Blood Center.

Year	Donors (N)	Repeat donors (N)	First-time donors (N)	Donations (N)	Repeat donors’ donations (N)	First-time donors’ donations (N)
2004	39,733	24,578	15,155	84,870	69,715	15,155
2005	42,173	20,388	21,785	90,915	69,130	21,785
2006	50,676	28,992	21,684	92,583	70,899	21,684
2007	52,975	26,357	26,618	92,284	65,666	26,618
2008	59,706	28,291	31,415	97,845	66,430	31,415
2009	60,019	41,679	18,340	94,326	75,986	18,340
2010	72,663	50,124	22,539	107,204	84,665	22,539
2011	59,615	36,581	23,034	87,971	64,937	23,034
2012	56,332	33,410	22,922	88,749	65,827	22,922
2013	37,776	22,170	15,606	61,254	45,648	15,606
2014	39,915	27,225	12,690	66,147	53,457	12,690
2015	45,525	25,148	20,377	70,774	50,437	20,337
2016	47,109	32,919	14,190	73,652	59,462	14,190
2017	44,890	31,538	13,352	67,817	54,465	13,352
2018	45,648	32,488	13,160	69,177	56,017	13,160
**Total**	754,755	461,888	292,867	1,245,568	952,741	292,827

Absolute numbers of first-time and repeat donors, and donations made by them in the National Blood Center of Lithuania from 2004 to 2018.

N-absolute number.

Each repeat donor performed 2.06 donations per year on average (the average inter-donation interval (IDI) was 177.66 days) in the National Blood Center of Lithuania. Over the 15-year period, the IDI increased from 107.65 days in 2005 to 211.69 days in 2018 (β = 5.80, 95% CI 3.037–8.563, p = 0.001).

All the donations included in this study were tested for markers of HBV, HCV and HIV infections. 0.78% of all donations (9,730 out of the 1,245,568) were confirmed to be positive for the serological or NAT tests during the study period and remained positive after confirmatory testing: 418 were positive for HIV(228 positive donations were made by first-time donors and 190 were made by repeat donors), 2,984 were positive for HBV (2,486 positive donations were made by first-time donors and 498 were made by repeat donors) and 6,328 were positive for HCV (4,786 positive donations were made by first-time donors and 1,542 were made by repeat donors).

As mentioned previously, results of NAT testing only since 2012 were included. In the study, 71 out of 1,489,238 donations tested with a NAT assay (0.005%) for the period from 2012 to 2018 were positive and remained that way after confirmatory testing. The NAT tested donations were as follows: only one HIV positive donation (from a repeat donor); 22 positive donations for HBV (11 positive donations were made by first-time donors and 11 were made by repeat donors); and 48 positive donations for HCV (13 positive donations were made by first-time donors and 35 were made by repeat donors).

**Fig 1:**
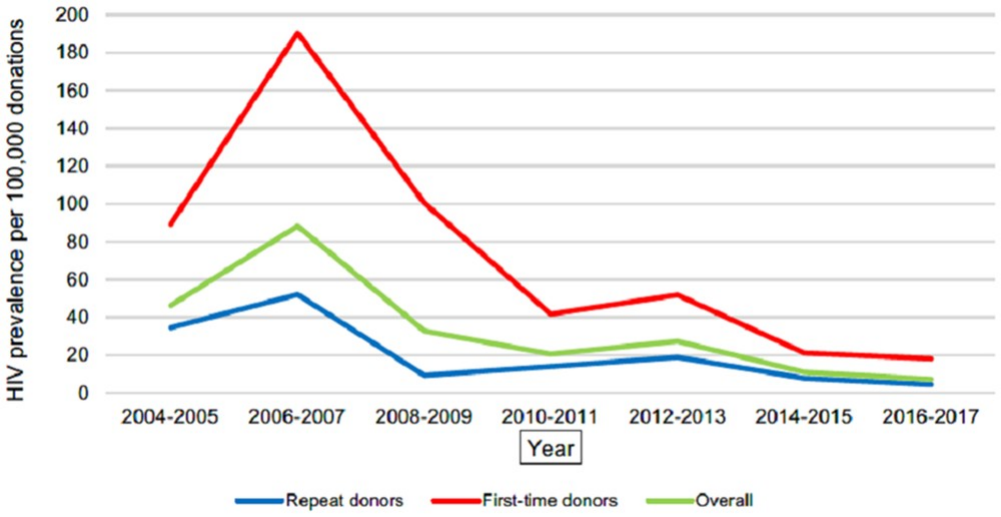
HIV prevalence per 100,000 donations. Annual trends of HIV prevalence per 100,000 donations in the National Blood Center of Lithuania from 2005 to 2017.

**Fig 2:**
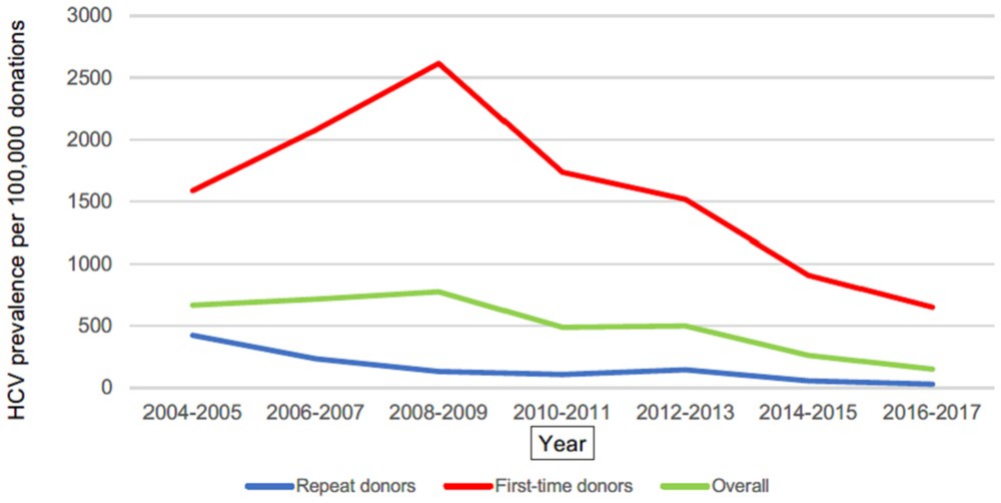
HCV prevalence per 100,00 donations. Annual trends of HCV prevalence per 100,000 donations in the National Blood Center of Lithuania from 2004 to 2017.

**Fig 3:**
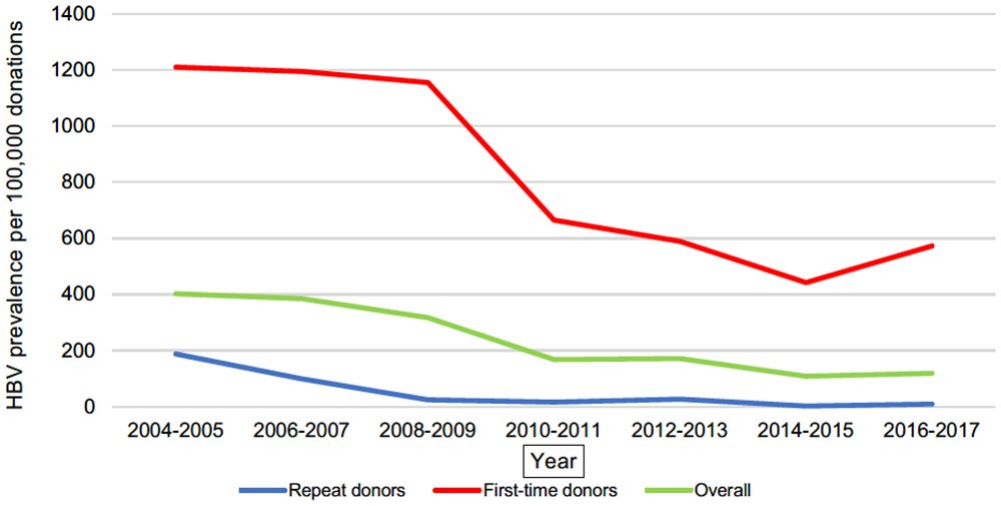
HBV prevalence per 100,000 donations. Annual trends of HBV prevalence per 100,000 donations in the National Blood Center Lithuania from 2004 to 2017.

The highest overall prevalence was evaluated for HCV in year 2008–2009–777,43 per 100,000 donations. It steadily decreased until year 2016–2017 and reached 151,98 per 100,000 donations. The overall prevalence of HBV during study period decreased from 402.76 per 100,000 donations (2004–2005 period) to 108.09 per 100,000 donations (2014–2015 period); the overall prevalence of HIV decreased from 88.17 per 100,000 donations (2006–2007 period) to 7.07 per 100,000 donations (2016–2017 period). During the study period, there was a statistically significant downward trend in the prevalence rate of HIV (β -4.61, 95% CI -7.02 to -2.20, p = 0.001), HBV (β -26.36, 95% CI -32.73 to -19.98, p<0.0001) and HCV (β -49.81, 95% CI -65.64 to -33.98, p<0.0001). Figs 1–3 present decreasing prevalence in 2-year intervals. A 16.0 times higher prevalence was noticed in donations made by first-time donors compared to the donations made by repeat donors for HBV infection; a 9.7 times higher prevalence was noticed in donations made by first-time donors compared to the donations made by repeat donors for HCV infection; and a 3.6 times higher prevalence was noticed in donations made by first-time donors compared to the donations made by repeat donors for HIV infection.

The average incidence of HBV during the 15-year study period was 349.59 per 100,000 person years and it decreased from 2730.08 per 100,000 person years in 2005 to 7.20 per 100,000 person years in 2014. The average incidence of HCV was 390.21 per 100,000 person years (it steadily decreased from 2310.18 per 100,000 person years in 2005 to 31.71 per 100,000 person years in 2017). The average incidence of HIV was 50.53 per 100,000 person years (it decreased from 235.43 per 100,000 person years in 2005 to 6.08 per 100,000 person years in 2016) (Table 3).

**Table 3:** Annual incidence rates in donors and residual risk of HIV, HCV and HBV transmission.

**HIV infection**					
**Year**	**Number of RD donors**	**Incidence per 100,000** **person years in RD**	**Residual risk per 1,000,000 donations for RD**	**Residual risk per 1,000,000 donations for FTD**	**Overall residual risk per 1,000,000 donations**
2004	39733	-^1^	-^1^	-^1^	-^1^
2005	42173	235.43	135.45	406.36	200.37
2006	50676	162.11	93.27	279.81	136.96
2007	52975	91.06	52.39	157.17	82.61
2008	59706	35.35	15.49	46.48	25.44
2009	60019	7.20	3.16	9.47	4.38
2010	72663	31.92	13.99	41.98	19.88
2011	59615	13.67	5.99	17.97	9.13
2012	56332	38.91	4.26	12.79	6.47
2013	37776	36.08	3.95	11.86	5.97
2014	39915	11.02	1.21	3.62	1.67
2015	45525	19.88	2.18	6.54	3.43
2016	47109	6.08	0.67	2.00	0.92
2017	44890	9.51	1.04	3.13	1.45
2018	45648	9.23	1.01	3.04	1.40
Average	50317	50.53	23.86	71.59	35.72
**HCV infection**					
**Year**	**Number of RD donors**	**Incidence per 100,000** **person years in RD**	**Residual risk per 1,000,000 donations for RD**	**Residual risk per 1,000,000 donations for FTD**	**Overall residual risk per 1,000,000 donations**
2004	39733	488.24	802.59	2407.77	1089.22
2005	42173	2310.18	3797.56	11392.68	5617.50
2006	50676	655.35	1077.29	3231.88	1581.92
2007	52975	508.40	835.73	2507.20	1317.84
2008	59706	325.19	534.56	1603.69	877.82
2009	60019	239.93	394.40	1183.21	547.77
2010	72663	147.63	242.69	728.06	344.73
2011	59615	246.03	404.43	1213.30	616.22
2012	56332	362.17	29.77	89.30	45.14
2013	37776	198.47	16.31	48.94	24.62
2014	39915	102.85	8.45	25.36	11.70
2015	45525	127.25	10.46	31.38	16.47
2016	47109	75.94	6.24	18.73	8.65
2017	44890	31.71	2.61	7.82	3.63
2018	45648	33.86	2.78	8.35	3.84
Average	50317	390.21	544.39	1633.18	807.14
**HBV infection**					
**Year**	**Number of RD donors**	**Adjusted incidence per 100,000** **person years in RD**^2^	**Residual risk per 1,000,000 donations for RD**	**Residual risk per 1,000,000 donations for FTD**	**Overall residual risk per 1,000,000 donations**
2004	39733	172.96	199.02	597.05	270.09
2005	42173	2730.08	3141.47	9424.40	4646.98
2006	50676	1217.76	1401.25	4203.76	2057.63
2007	52975	191.57	220.44	661.32	347.60
2008	59706	253.90	292.16	876.49	479.77
2009	60019	110.83	127.53	382.59	177.12
2010	72663	114.30	131.52	394.57	186.83
2011	59615	107.51	123.71	371.14	188.50
2012	56332	146.28	68.13	204.39	103.33
2013	37776	42.37	19.73	59.20	29.79
2014	39915	7.20	3.35	10.06	4.64
2015	45525	7.65	3.56	10.69	5.61
2016	47109	19.26	8.97	26.91	12.43
2017	44890	41.86	19.49	58.48	27.17
2018	45648	20.34	9.48	28.43	13.08
Average	50317	345.59	384.65	1153.97	570.04

Incidence rates of HIV, HCV and HBV per 100,000 person years and residual risk of transfusing an infected with transfusion transmitted viruses’ blood/its component per 1 million donations in the National Blood Center of Lithuania from 2004 to 2018.

FTD—first-time donors of blood or its components.

RD—repeat donors of blood or its components.

^1^HIV incidence rate and residual risk for year 2004 was not calculated due to the absence of seroconverting donors for HIV in that year.

^2^ An adjustment factor, described in ‘Statistical analysis’ part of this article, was used for the residual risk of HBV calculation.

Average overall residual risk of HBV, HCV and HIV during study period accordingly was 570.04, 807.14 and 35.72 per 1,000,000 donations (Table 3). During the study period, there was statistically significant downward trend in the residual risk for every TTV, including the overall residual risk and the residual risk for repeat donor donations and first-time donor donations (Table 4).

**Table 4:** Trends of HBV, HCV and HIV transmission residual risk.

TTV		β	95% CI	p
**HIV**	Repeat donors	-7.39	-11.54 –-3.24	0.002
	First-time donors	-22.17	-34.63 –-9,71	0.002
	Overall	-11.04	-17.14 –-4.95	0.002
**HBV**	Repeat donors	-103.69	-197.27 –-10.10	0.032
	First-time donors	-311.07	-591.82 –-30.31	0.032
	Overall	-153.27	-291.56 –-14.98	0.032
**HCV**	Repeat donors	-141.33	-239.73 –-42.93	0.008
	First-time donors	-423.99	-719.19 –-128.79	0.008
	Overall	-208.2	-354.29 –-62.12	0.009

Trends of HBV, HCV and HIV transmission residual risk per 1,000,000 donations in the National Blood Center of Lithuania from 2004 to 2018.

The average HBV and HCV residual risk during the period when antigen EIA/CLIA screening assay was used was statistically significantly higher than average residual risk of the period of ID-NAT assays usage (p<0.0001). The average HIV residual risk during the period when antibody EIA/CLIA screening assay was used was statistically significantly higher than average HIV residual risk of the period of Combo EIA/CLIA usage (p = 0.008) and period of ID-NAT assays usage (p<0.0001) (Table 5).

**Table 5:** HIV, HCV and HBV transmission residual risk and residual risk trends by period of screening assay.

TTV	Years	Overall residual risk per 1,000,000 (95%CI)	Trends of residual risk	
			β (95%CI)	p
HIV	2005–2007^1^	139.98 (-6.43–286.39)	-58.88 (-92.10 –-25.65)	0.028
	2008–2011	14.71 (-0.66–30.07)	-3.35 (-23.70–17.01)	0.553
	2012–2018	3.04 (0.91 to 5.18)	-0.89 (-1.57 –-0.21)	0.020
HCV	2004–2011	1499.13 (65.93–2932.33)	-395.44 (-972.27–181.39)	0.144
	2012–2018	16.29 (2.70–29.89)	-6.03 (-9.65 –-2.42)	0.008
HBV	2004–2011	1044.32 (-282.42 to 2371.05)	-337.87 (-890.09–214.34)	0.185
	2012–2018	28.01 (-4.01 to 60.02)	-9.58 (-24.35–5.19)	0.156

Residual risk of HIV, HCV and HBV transmission per 1,000,000 donations and its trends divided into periods by the corresponding screening assays used in the National Blood Center of Lithuania from 2004 to 2018.

^1^The residual risk for HIV for year 2004 was not included due to the absence of converting donors for HIV for that year.

Statistically significant downward trend of residual risk was noticed for HIV in the periods from 2005 to 2008 and from 2012 to 2018; for HCV—in the period from 2012 to 2018 when ID-NAT assay was used for testing. Although, HBV residual risk reduced during periods of antigen EIA/CLIA and ID-NAT assays usage, trends were not statistically significant (Table 5).

## Discussion

In order to develop efficient policies and evidence-based donor screening strategies, data about the prevalence, incidence and residual risk of HBV, HCV and HIV transmission is necessary. The residual risk of transmitting TTVs during blood donations differs across the world due to the differences in demographic, socio-economic characteristics and the various TTV risk factors, so population-based surveys play an important role. To our knowledge, the residual risk of missing a contamination of the blood or its components has never been evaluated in Lithuanian blood donors, which makes this survey both scientifically and practically important.

All blood and blood component’s donations undergo mandatory testing for HBV, HCV and HIV using antigen/antibody assays in the National Blood Center of Lithuania. All seronegative donations are tested for HBV, HCV and HIV with NAT since 2005. It allowed for us to compare residual risk before and after introduction of NAT testing. Confirmatory tests are performed on all initially serologically or NAT positive donations for an investigation of particular viral agent.

Since the adoption of restrictive criteria for donor selection, together with the implementation modern laboratory tests for blood screening, it is difficult and expensive to directly evaluate the residual risk of TTV transfusions in practice. Fortunately, mathematical models for the purpose of calculating the TTV transfusion residual risk can be applied. We chose the incidence/window-period model as suggested by the WHO because this model presents clear advantages. Firstly, incidence/window-period model allows an approximate estimation of the residual risk based on limited data. Also, by using this model, an evaluation of the residual risk in repeat donors can be extrapolated for the corresponding risk in first-time donors, for which a calculation of the incidence rates is mostly impossible. Finally, this model allows for a comparison of the data across the world if an unified model is used to evaluate residual risk in different countries [17].

The prevalence rate of HBV, HCV and HIV was found to be steadily decreasing (statistically significant downward trends evaluated for all three viruses presented in Figs 1–3) in the National Blood Center of Lithuania during the study period. An increase in the prevalence for HIV and HCV was noticed accordingly in period 2006–2007 and periods 2006–2007, 2008–2009 compared to the rates in 2004–2005. This can be explained by the fact that NAT testing was introduced in the National Blood Center of Lithuania in 2005, and this has resulted in improved testing quality which led to more accurate statistics.

HCV has demonstrated the highest prevalence rates through all study period. This shows that this infection was (especially before the start of the new treatment strategy in Lithuania since 2015) and still is difficult to control. Blood or its components are donated by healthy citizens aged from 18 to 65 years in Lithuania, so it is possible that HCV infection is not sufficiently controlled in the general population. Especially because there is no vaccination against this infection.

The results of this study highly support the strategy where the safest blood is collected from repeat, voluntary and non-remunerated blood donors [8, 10, 11]. As per the results of our survey, much higher prevalence rates were found in first-time blood donors compared to repeat donors for HBV, HCV and HIV infections (respectively, there was a 16.0, 9.7 and 3.6 times higher prevalence rates in first-time donors than in repeat donors).

The average incidence of HBV, HCV and HIV in repeat donors, respectively, was 349.59 per 100,000 person years, 390.21 per 100,000 person years and 50.53 per 100,000 person years. HCV was the agent for which the highest incidence was recorded in almost all the years of the study period (except 2005–2006 and 2017), so the overall residual risk trend was mostly affected by the rates of this virus (Table 3). This once again supports idea that HCV infection control in Lithuania requires improvement. It is worth mentioning that incidence rates evaluated in blood donors of this survey were much higher, especially for HBV and HCV infections, compare to the ones in general population of Lithuania and European Union/European Economic Area countries for period from 2012 to 2017 (Table 6). This can partly be explained by an epidemiological characteristic of these infections and statistical inaccuracies they might result in. Rates of spontaneous viral clearance in people infected with acute HBV or HCV varies from 5% to 25%; for the rest—acute infections become chronic. Chronic HBV, HCV infections often remain silent and undiagnosed for years, because “silent carriers” usually do not seek medical attention and asymptomatic cases of HBV and HCV remain undescribed in official statistics. Also, in Lithuania, as in many other EU/EEA countries, only acute hepatitis cases were compulsorily registered until year 2019. Almost three times more cases of hepatitis were registered in the first half of year 2019, after the introduction of chronic hepatitis registration in Lithuania, than in the same period last year. Higher HBV and HCV incidence rates in blood donors, who undergo mandatory screening, compare to the general population can indicate lack of effective national screening program for these viruses. Even WHO has agreed that the public health threat of viral hepatitis has long been underestimated and recently in the ‘2030 Agenda for Sustainable Development’ called for international action to combat viral hepatitis, with the aim of drastically reducing the disease burden by 2030. One of the barriers for achieving this goal is incomplete local surveillance systems [21–23].

**Table 6:** Cases of HBV, HCV and HIV infections reported in Lithuania and Europe.

Year	Acute HBV infection^4^				Acute HCV infection^5^				HIV infection			
	Lithuania^1^		EU/EEA^2^		Lithuania^1^		EU/EEA^2^		Lithuania^1^		EU/EEA^2^	
	N	Rate^6^	N	Rate^6^	N	Rate^6^	N	Rate^6^	N	Rate^6^	N	Rate^6^
**2012**	23.0	0.77	2,.798	0.8	40.0	1.34	509.0	0.6	150.0	5.36	160.0	5.3
**2013**	35.0	1.18	2.896	0.7	59.0	1.99	569.0	0.5	168.0	5.98	177.0	6.0
**2014**	26.0	0.89	2.667	0.6	34.0	1.16	458.0	0.6	136.0	4.81	141.0	4.8
**2015**	32.0	1.1	2.505	0.6	23.0	0.79	346.0	0.4	164.0	5.4	157.0	5.4
**2016**	32.0	1.12	2.529	0.6	16.0	0.56	813.0	0.4	207.0	7.46	214.0	7.4
**2017**	14.0	0.88	2.486	0.6	25.0	0.88	861.0	0.3	263.0	9.3	91.0	9.1
**2018**	13	0.89	-^3^	-^3^	25	0.89	-^3^	-^3^	160	5.71	-^3^	-^3^
**Average**	**40.4**	**1.3**	**2.686**	**0.7**	**39.2**	**1.2**	**501.0**	**0.5**	**196.8**	**5.7**	**160.0**	**5.9**

Absolute numbers and rates of diagnosed and reported acute HBV, acute HCV and HIV cases per 100, 000 person in general population of Lithuania and European Union/European Economic Area countries from 2012 to 2018 years.

EU/EEA—European Union/European Economic Area countries.

N—absolute number of cases.

^1^Absolute numbers and rates for the HBV, HCV and HIV cases diagnosed in Lithuania were collected from the statistical forms of the Centre for Communicable Diseases and AIDS of Lithuania and Institute of Hygiene (Lithuania) for the period from 2012 to 2018.

^2^ Absolute numbers and rates for the HBV, HCV and HIV cases diagnosed in the EU/EEA were collected from the annual surveillance reports prepared by the European Centre for Disease Prevention and Control for the period from 2012 to 2017.

^3^ Annual surveillance report of European Centre for Disease Prevention and Control for year 2018 was not published during preparation of this article.

^4^Acute HBV infection cases were defined by the detection of the IgM core antigen-specific antibody (anti-HBc IgM) OR detection of the hepatitis B surface antigen (HBsAg) and previous negative HBV markers less than six months ago OR detection of the hepatitis B nucleic acid (HBV-DNA) and previous negative HBV markers less than six months ago.

^5^Acute HCV infection cases were defined by a recent HCV seroconversion (prior negative test for hepatitis C in the last 12 months) OR detection of the hepatitis C virus nucleic acid (HCV RNA) OR a hepatitis C virus core antigen (HCV-core) in the serum/plasma and no detection of the hepatitis C virus antibody (negative result).

^6^Notification rates were calculated per 100,000 members of the population annually.

The highest incidence rate for all three viruses was recorded in 2005. In the same year, the highest overall residual risk for TTVs was recorded (for HBV 4646.98 per 1,000,000 donations; for HCV, 5617.50 per 1,000,000 donations; and for HIV, 200.37 per 1,000,000 donations). This peak in the incidence could be explained by the implementation of new laboratory tests (antibody/antigen based assays together with NAT) at the National Blood Center of Lithuania, resulting in better diagnostics for new cases and more accurate statistical data. However, residual risk for period from 2004 to 2011, should be carefully examined. NAT testing was introduced in 2005 at the National Blood Center of Lithuania, but due to specific of statistical forms results of NAT testing were available only since 2012. So, vDWP of serological tests was used for calculation of residual risk for period from 2004 to 2011 (Table 3), which could result in increase of residual risk.

In year 2012, when we included NAT test results of blood donors, increase in incidence was evaluated for all three viruses. These increase in incidence supports an importance of modern laboratory test introduction in order to identify as much cases of viruses in donations as possible. However, overall residual risk decreased for all three viruses in year 2012. Also, average HBV, HCV and HIV residual risk during the period 2004–2001 when antigen EIA/CLIA screening assay was used was statistically significantly higher than average residual risk of the period 2012–2018 of ID-NAT assays usage (p<0.0001). The average HIV residual risk during the period when antibody EIA/CLIA screening assay was used was statistically significantly higher than average HIV residual risk of the period of Combo EIA/CLIA usage (p = 0.008) as well (Table 5). This once again shows how modern laboratory tests results in more accurate blood screening results and lowered residual risk of TTV transmission.

One of the most surprising findings of this study was an increase of the incidence and residual risk for HBV infection in 2016–2018 compared to the two previous years. For this period transmission residual risk of HBV was higher than the one evaluated for HCV (virus that has demonstrated the highest incidence and residual risk almost through all the study). It is worth mentioning that reduce of HBV transmission residual risk during periods of antigen EIA/CLIA and ID-NAT assays usage was not statistically significant. These findings can indicate difficulties of controlling spread of HBV in general population. A significant proportion of HBV cases, as well as HCV, remain asymptomatic and members of general population do not seek medical help until manifestation of infection.

Throughout the 15 years of the study period, residual risk of HBV, HCV and HIV transfusion was steadily decreasing in the National Blood Center of Lithuania. During the study period, statistically significant downward trends were evaluated in the residual risk for every TTV, including the overall residual risk (Table 4). Although, it is difficult to compare incidence rates and residual risk between various countries, because of differences in screening strategies and collection of routine statistics, we can see that residual risk in Lithuania is much higher compare to other countries [12, 24, 25]. This can partly be explained by the fact that incidence rates evaluated in blood donors of this survey were much higher compare to ones in European Union/European Economic Area countries (Table 6). Residual risk rates can be influenced by multicomplex donor testing at the National Blood Center of Lithuania and difficulties in choosing the corresponding vDWP as well. Besides, vDWP for serological testing was used from 2004 to 2011, although NAT testing has been already implemented in the National Blood Center of Lithuania for this period.

As mentioned before, monitoring of the TTV transmission residual risk by mathematical models is useful in practice for the detecting main risk groups and the targets of action, but results of this study could be affected by some disadvantages:

1. Data from the archives of the National Blood Center of Lithuania, that did not include sociodemographic information (such as age group and sex) of individual donors has been analysed. Donor’s sociodemographic characteristics are known to be closely related to the epidemiology of HBV, HCV and HIV infections and are very important for interpretation of TTVs epidemiological patterns. Variations in TTVs’ incidence, prevalence and residual risk can be affected by changing donor’s characteristics and age cohort effects. Lack of sociodemographic characteristics of individual donors could be considered as a major limitation of this study.
2. NAT tests results were included in the calculation of residual risk only starting from 2012, because of specifics of routine statistic collection in Lithuania. This could have had an impact on the data accuracy for the period from 2004 to 2011. For this period vDWP of serological testing was used and this could result in higher estimated residual risk than the actual one is.
3. Disadvantages of the incidence/window period model include:
  - The model used in this study is highly depended on the length of the DWP, which can be affected by the differences of manufacturers presenting tests in the same category. Also, it can be affected the donors’ screening strategies used in different donation centres.
  - For evaluating the residual risk, new cases are the most important, but they are affected by the DWP. Seroconvertive blood donors are used in this model, as there is the biggest possibility that they have donated a positive donation within the DWP. In the WHO model, we used, the presumption is that all repeat donors have made at least two donations in the year when residual risk was calculated. Unfortunately, this presumption can only be checked by a direct follow-up of individual donors.
  - The incidence rate of first-time donors cannot be directly calculated by using this model. An adjustment factor based on the results of studies conducted in regions with different characteristics is used, because the incidence between first-time and repeat donors is unknown in most countries [17].

Due to limitations of this study mentioned above, the data obtained cannot be assumed to be exact, but it can illustrate approximate magnitude of the problem. Modern and up to date laboratory tests and donor selection/testing strategies are used at the National Blood Center of Lithuania, but incidence rates and residual risk of HBV, HCV and HIV remains high compare to general population of Lithuania and other countries. This can be a warning indicator of actual HBV, HCV and HIV spread in Lithuania. As mentioned previously, the large proportion of HBV and HCV infections remain asymptomatic and the real risk could be devalued. Even WHO recognized viral hepatitis as a “largely ignored” health problem in 2016, which led to a World Health Assembly resolution to eliminate viral hepatitis as a major public health problem by 2030 [23]. The world now has the tools to prevent hepatitis B and cure hepatitis C—a new direct-acting antivirals which can cure 95 percent of chronic infections. Though these drugs are unlikely to reach all chronically infected people. So, the keys to elimination of hepatitis are identification of people who have the disease and making sure that they are appropriately linked to care. One of the biggest challenges in fighting viral hepatitis is poor national screening strategies. Prevalence, incidence rates and residual risk of TTV transfusion in Lithuanian blood donors corresponds with WHO ideas and indicates need of future research of HBV, HCV, HIV in general population, as well as the need of national screening program. Especially now, when there are more and more possibilities to fight these infections.

## Supporting information

S1 File Summarized data of blood and its components donors, donations made by them and numbers of HBV, HCV and HIV markers confirmed at the National Blood Center of Lithuania, 2004–2018. (PDF)
