# Supplementary material for: Prevalence, incidence and residual risk of transfusion transmitted viruses (HBV, HCV and HIV infections) in Lithuanian blood donors from 2004 to 2018: The incidence/window-period model study
Source: PLoS One. 2021 Feb 19;16(2):e0246704. doi: 10.1371/journal.pone.0246704 (PMC7894937; doi:10.1371/journal.pone.0246704)
Supplement: S1 File — (PDF) [file pone.0246704.s001.pdf]

Number	Year	Donations_total	Donations_RD	Donations_FTD	Repeat_donors	FT_donors	Donors_total	HIV_RD_pos	HIV_FTD_pos	HBsAg_RD_pos	HBsAg_FTD_pos	anti_HCV_RD_pos	anti_HCV_FTD_pos	NAT_HIV_RD_tests	NAT_HIV_RD_pos	NAT_HIV_FTD_tests	NAT_HIV_FTD_pos	NAT_HBV_RD_tests	NAT_HBV_RD_pos	NAT_HBV_FTD_tests	NAT_HBV_FTD_pos	NAT_HCV_RD_tests	NAT_HCV_RD_pos	NAT_HCV_FTD_tests	NAT_HCV_FTD_pos	HIV_DWP-ID-NAT	HBV_VDWP ID-NAT	HCV_DWP ID-NAT
1	2004	84870	69715	15155	24578	15155	39733	0	31	16	299	120	380		0		0		0		0		0		0	21	42	60
2	2005	90915	69130	21785	20388	21785	42173	48	2	245	148	471	206		0		0		0		0		0		0	21	42	60
3	2006	92583	70899	21684	28992	21684	50676	47	43	117	233	190	382		0		0		0		0		0		0	21	42	60
4	2007	92284	65666	26618	26357	26618	52975	24	49	17	344	134	621		0		0		0		0		0		0	21	42	60
5	2008	97845	66430	31415	28291	31415	59706	10	11	23	345	92	633		0		0		0		0		0		0	16	42	60
6	2009	94326	75986	18340	41679	18340	60019	3	39	12	230	100	669		0		0		0		0		0		0	16	42	60
7	2010	107204	84665	22539	50124	22539	72663	16	8	14	174	74	437		0		0		0		0		0		0	16	42	60
8	2011	87971	64937	23034	36581	23034	59615	5	11	10	129	90	354		0		0		0		0		0		0	16	42	60
9	2012	88749	65827	22922	33410	22922	56332	13	16	21	146	105	377	65827	0	22922	0	65793	4	22989	4	65793	16	22989	8	4	17	3
10	2013	61254	45648	15606	22170	15606	37776	7	4	3	77	38	198	45648	1	15606	0	46440	2	14818	0	46459	6	14822	2	4	17	3
11	2014	66147	53457	12690	27225	12690	39915	3	5	0	70	24	174	53457	0	12690	0	50192	1	16020	1	50176	4	15755	0	4	17	3
12	2015	70774	50437	20337	25148	20377	45525	5	2	1	74	24	127	50437	0	20337	0	52976	0	17133	1	52953	8	17080	0	4	17	3
13	2016	73652	59462	14190	32919	14190	47109	2	4	1	83	24	92	59462	0	14190	0	58429	2	14838	2	58429	1	14838	1	4	17	3
14	2017	67817	54465	13352	31538	13352	44890	3	1	6	72	10	85	54442	0	13352	0	54442	0	13157	1	54442	0	13157	2	4	17	3
15	2018	69177	56017	13160	32488	13160	45648	3	2	1	51	11	38	55938	0	12978	0	55938	2	12978	2	55938	0	12978	0	4	17	3
